# A position statement on the post gene-therapy rehabilitation of aromatic I-amino acid decarboxylase deficiency patients

**DOI:** 10.1186/s13023-024-03019-x

**Published:** 2024-01-18

**Authors:** Hui-Min Lee, Saadet Mercimek-Andrews, Gabriella Horvath, Diana Marchese, Richard E. Poulin, Alexis Krolick, Kati-Lyn Tierney, Jasmine Turna, Judy Wei, Wuh-Liang Hwu

**Affiliations:** 1 Department of Medical Genetics and Pediatrics, National Taiwan University Hospital and National Taiwan University College of Medicine, No. 8, Chung-Shan South Road, Taipei, 100226 Taiwan; 2 Department of Physical Therapy and Assistive Technology, National Yang Ming Chiao Tung University, Linong St. Beitou Dist, No. 155, Sec. 2, Taipei, 112304 Taiwan; 3 Department of Medical Genetics, University of Alberta, 8613 114 Street, Edmonton, AB T6G 2H7 Canada; 4 Division of Biochemical Genetics, Department of Pediatrics, University of British Columbia, 4480 Oak Street, Vancouver, BC V6H 3V4 Canada; 5 Department of Pediatric Rehabilitation, Children’s Mercy Hospital, 2401 Gillham Road, Kansas City, MO 64108 USA; 6 Department of Pediatrics, University of Missouri–Kansas City, 2411 Holmes Street, Kansas City, MO 64108 USA; 7 Thai-Chinese International School, 101/177 Moo 7 Soi Mooban Bangpleenives, Prasertsin Road, Bangplee Yai, Samut Prakan, 10540 Thailand; 8 PTC Therapeutics Inc, 100 Corporate Ct #2400, South Plainfield, NJ, 07080 USA; 9 Texas Children’s Hospital, 6621 Fannin Street, Houston, TX 77030 USA; 10 Ruamrudee International School, 6 Soi Ramkhamhaeng 184, Khwaeng Min Buri, Min Buri, Bangkok, 10510 Thailand; 11 Center for Precision Medicine, China Medical University Hospital, 2 Yude Road, 404 Taichung City, Taiwan

**Keywords:** Gene therapy, AADC deficiency, Physical therapy, Neurotransmitter disorder, Rehabilitation outcomes, Gross motor development, Fine motor development

## Abstract

Aromatic l-amino acid decarboxylase (AADC) deficiency is a rare genetic disorder of monoamine neurotransmitter synthesis that presents with a range of symptoms, including motor dysfunction and limited attainment of developmental motor milestones. The approval of eladocagene exuparvovec, a gene therapy for AADC deficiency with demonstrated efficacy for motor improvements, now expands the range of motor outcomes possible for patients with this disorder. However, recommendations and guidelines for therapy following treatment with gene therapy are lacking. To ensure patients can reach their full potential following treatment with gene therapy, it is essential they receive rehabilitation therapies designed specifically with their impairments and goals in mind. Therefore, we highlight specific rehabilitative needs of patients following gene therapy and propose a set of recommendations for the post-treatment period based on collective experiences of therapists, physicians, and caregivers treating and caring for patients with AADC deficiency who have been treated with gene therapy. These recommendations include a focus on periods of intensive therapy, facilitating active movements, training for functional abilities, cognitive and communication training, parent/caregiver empowerment, collaboration between therapists and caregivers to develop in-home programs, and the incorporation of supplemental forms of therapy that patients and their families may find more enjoyable and engaging. Many of these rehabilitative strategies may be employed prior to gene therapy. However, these recommendations will be valuable for therapists, caregivers, and wider treatment teams as they prepare for the post-treatment journey with these patients. Furthermore, the considerations and recommendations presented here may prove beneficial outside the AADC deficiency community as gene therapies and other treatments are developed and approved for other rare diseases.

## Background

Aromatic l-amino acid decarboxylase (AADC) deficiency is a rare genetic disorder of neurotransmitter biosynthesis resulting from pathogenic variants in the dopa decarboxylase gene (OMIM #608,643) [1] encoding the AADC enzyme [2] (EC 4.1.1.28) [3, 4]. As AADC converts l-3,4-dihydroxyphenylalanine (l-DOPA) to dopamine, and 5-hydroxytryptophan (5-HTP) to serotonin, a deficiency of the enzyme results in severe deficits of dopamine, serotonin, norepinephrine, and epinephrine [3, 4]. A deficiency of these neurotransmitters results in a wide spectrum of symptoms, including, but not limited to, motor dysfunction and motor arrest, cognitive impairments, dystonia, oculogyric crises, failure to thrive, seizures, and autonomic dysfunction [3–6]. Symptom severity can vary, but literature reviews and surveys of patients with AADC deficiency have found that most known cases show profound motor impairments with no or very limited gross and fine motor milestone development [4, 7]. These milestones typically develop sequentially, and the delayed development of earlier milestones has major consequences for patients with AADC deficiency. Patients may also experience sequelae arising from hypotonia and akinesia, including feeding difficulties, gastrointestinal reflux, aspiration pneumonias, joint contractures, scoliosis, and hip dysplasia [4, 5, 7, 8]. Diagnosis of AADC deficiency can be difficult, as patients may present with developmental delay or may be mistakenly diagnosed with epilepsy/seizures which is often more likely in these individuals to be oculogyric crisis or other movement disorders [4, 9].

In July 2022, the European Commission approved the first gene therapy for the treatment of AADC deficiency, eladocagene exuparvovec, in the European Union member states and Iceland, Liechtenstein, Northern Ireland, and Norway [10]. Authorization in the United Kingdom by the Medicines and Healthcare Products Regulatory Agency followed in November 2022 [11].

Eladocagene exuparvovec consists of a recombinant adeno-associated viral vector serotype 2 (AAV2) containing the complementary DNA encoding the healthy human AADC enzyme that is infused directly into the putamen via stereotactic neurosurgical delivery. Once delivered to the putamen, eladocagene exuparvovec drives the expression of the AADC enzyme, restoring the enzymatic pathway and production of dopamine [12].

Three clinical trials examined efficacy and safety of eladocagene exuparvovec in patients with AADC deficiency, with data published for 26 patients with at least 12 months of follow-up. Before treatment, all patients lacked any developmental milestones, including age-appropriate head control. All patients treated with eladocagene exuparvovec showed significant increases on both the Peabody Developmental Motor Scales–2nd Edition (PDMS-2) and Alberta Infant Motor Scale (AIMS) at 1, 2, and 5 years post-treatment compared to baseline. Gross and fine motor improvements were observed as early as 3 months following gene therapy. Additionally, 3 patients gained the ability to walk without assistance within 3 years after treatment. Among 5 patients with longer-term follow-up (6–10 years after treatment), PDMS-2 and AIMS scores remained markedly elevated compared to baseline. Patients also showed improved cognitive and language ability, as measured by the Comprehensive Developmental Inventory for Infants and Toddlers and the Bayley Scales of Infant and Toddler Development–3rd Edition (Bayley-III). Gene therapy was well tolerated; the most common treatment-emergent adverse events were fever and mild to moderate dyskinesia, which resolved within a few months following gene therapy [13]. Together, these results demonstrate the expanded opportunity for motor improvements following gene therapy. Further improvements may also be observed from post-treatment data collected via a long-term patient registry [12].

The importance of rehabilitation therapy, particularly physical therapy, in management and treatment of patients with AADC deficiency has been acknowledged [4, 14], especially given the motor impairments and severe delay in developmental motor milestones common in the disorder [4, 7]. However, gene therapy for treatment of AADC deficiency was only recently authorized for use, and existing consensus guidelines for diagnosis and treatment of AADC deficiency do not provide clear recommendations for type, frequency, or duration of rehabilitation therapies following gene therapy [4]. Now that a gene therapy with demonstrated efficacy for AADC deficiency has been approved, there is an urgent need for development of post-treatment therapy guidelines to maximize improvements of motor function following gene therapy.

This article highlights specific rehabilitative needs of patients following gene therapy and proposes a framework based on experiences of therapists, physicians, and parents/caregivers involved in treating and caring for patients with AADC deficiency following gene therapy. Recent approval of eladocagene exuparvovec and the rarity of AADC deficiency have limited any formal investigations into the effectiveness of various therapy approaches. Therefore, these recommendations are based on collective professional and personal experiences as well as studies from other pediatric disorders presenting with motor impairments, such as cerebral palsy (CP), Rett syndrome, and others. It is important to acknowledge that patients are unique and will experience a various range of improvements following gene therapy. The guidelines that follow are focused primarily on rehabilitation and are intended to serve as recommendations and considerations based on expert opinion. Therapy regimens, including medications, should be developed in collaboration with the entire treatment team to best serve each patient.

## Main text

**Fig. 1:**
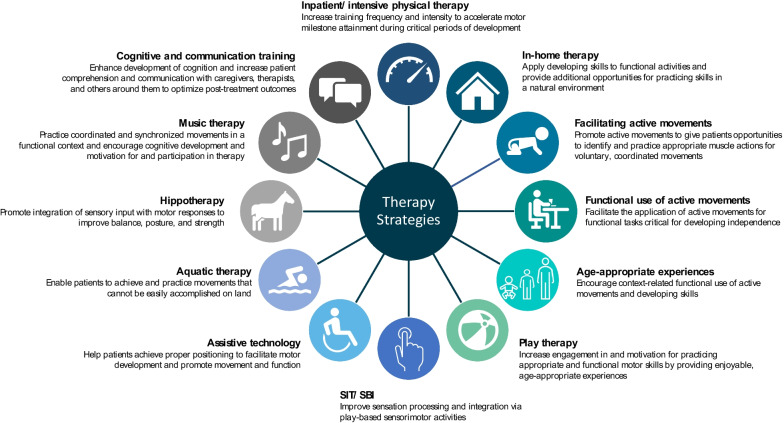
Overview of recommended therapy strategies for patients with AADC deficiency following treatment with gene therapy. Recommended therapy strategies and their primary intended goal for patients with AADC deficiency following treatment with gene therapy are displayed. The treatment team should work in collaboration with the patient/caregiver to develop a treatment plan that is most appropriate for each patient as they progress following gene therapy. *SBI* sensory-based interventions, *SIT* sensory integration therapy

Patients with AADC deficiency experience a broad spectrum of gross motor, fine motor, and cognitive impairments [3–5], and response to gene therapy varies among patients [13]. Based on published literature and expert opinion, we identified several therapy modalities that may be employed to benefit patients once medically stable following gene therapy administration, and in many instances, prior to gene therapy (Fig. 1). These strategies may include a focus on gross motor, fine motor, language, and cognitive development, but many have also been shown to improve social interactions, self-confidence, and motivation in patients with other pediatric disorders [15–20]. Patients receiving gene therapy have shown specific improvements in autonomic dysfunction, including reduction in hyperhidrosis, and hyperthermia, as per retrospective caregiver assessments. However, patients treated with gene therapy may still be prone to hypotension, hypoglycemia, and temperature instability [13]. As a result, extra caution should be taken during physical training, as these events are preventable.

### Therapy needs and considerations

#### Intensive and inpatient physical therapy

Due to the extent of motor impairments frequently observed in the disorder and the potential for accelerated skill acquisition after gene therapy, patients with AADC deficiency may benefit from a more intensive physical therapy regimen. Intensive regimens may help take advantage of developing motor control brain networks observed following intraputaminal rAAV2-hAADC gene therapy [21]. Intensive regimens typically consist of short bursts of intense practice for several hours daily, providing increased opportunity for motor learning. Bursts occur for 3 or 4 weeks and are followed by a period of normal activity and no therapy. Caregiver interviews and observations of patients with CP revealed improvements in strength, endurance, functional gains, and independence following intensive physical therapy regimens [22]. A review of intensive physical therapy and neurodevelopmental therapy (occurring > 3 times weekly) demonstrated improved scores on the Gross Motor Function Measure in children with CP. The beneficial effects of intensive therapy were stronger for children aged ≤ 2 years [23]. Another study in children with CP aged ≤ 2 years found motor improvement from intermittent intensive physical therapy may depend on patient baseline severity, with patients unable to sit without support experiencing greater improvements [24]. From the motor learning perspective, recommendations on training frequency and intensity used for patients with CP may be applied to patients with AADC deficiency. Since eladocagene exuparvovec gene therapy has been evaluated only in patients with limited to no attainment of basic motor milestones at baseline [12], following with intensive physical therapy is expected to be promising.

In addition to potentially greater motor improvements, frequency of intensive physical therapy allows short-term goals to be adapted daily and improves quality of time spent in physical therapy and the therapist/patient relationship. Rest periods between intensive bursts allow patients and families a more normal lifestyle without the stress of therapy sessions [22, 25]. Furthermore, physical therapy compliance in patients with CP improved when therapy sessions increased from 2 to 4 times weekly [25].

Another option for intensive physical therapy for patients with AADC deficiency following gene therapy is pediatric inpatient rehabilitation admission. In addition to improved motor outcomes for patients, an initial stay in an inpatient rehabilitation center may reduce stress on caregivers as they can learn and prepare for post-gene therapy care in a well-controlled environment. Inpatient rehabilitation also allows collaboration across the treatment team for necessary assistive devices, such as wheelchairs, adaptive strollers, and bath equipment. An alternative to inpatient rehabilitation, which may not be available to all patients due to limited accessibility, cost to families, distance to centers or other family rationale, is an outpatient model such as the Intensive Model of Therapy, which has been used to treat patients with CP [26]. These outpatient regimens can be given in ambulatory settings in the immediate periods after surgery and can be adapted to best suit needs of individual patients. Motor milestone data from clinical studies in patients with AADC deficiency demonstrate rapid milestone attainment following acquisition of the first motor milestone over the first year following gene therapy [27]. Hence, patients may benefit most from intensive bursts of therapy within this critical period. At the therapist’s discretion, intensive therapy may also be utilized to promote development of the first motor milestone.

#### In-home therapy

Following gene therapy, in-home therapy is recommended in different phases, including the early months when patients are learning basic motor skills and do not require more advanced equipment offered by treatment centers or clinics, as well as in a later period, when patients learn to apply or adapt their movements for daily functional activities at home. In-home therapy plays an important role in successful therapy outcomes in patients with CP by increasing the amount of time spent practicing motor skills and improving use of functional skills in natural environments [28–30]; it should continue as patients progress in motor development.

In-home therapy should also focus on routine-based intervention rather than only direct therapy. Incorporating in-home therapy with routine-based considerations can help caregivers learn how to integrate therapy throughout their daily routine and encourage their child to use developing skills in everyday activities. From our experiences, patients with AADC deficiency have difficulties transferring movements into functional use, even after gene therapy. This routine-based approach and integration of skills into daily activities is critical, as complex in-home therapy regimens that are difficult to incorporate into the family’s routine have been identified as barriers to compliance. Therapists can work with caregivers in many ways to improve compliance with at-home therapy, including demonstrating exercises; providing written instructions with feedback, incentives, and reminders; and helping caregivers identify ways to incorporate skills into their daily routine [30]. Therapists should also strive for a collaborative relationship with the caregiver throughout an in-home program, as partnership home programs where both parties are involved in developing therapy goals, activities, and expectations can be more motivating and achievable [31]. Further support for collaborative programs can be seen in children with CP, where family-centered therapy services have been associated with increased family and recreational participation, as well as improved motor outcomes [32].

With the increased utilization of telehealth [33], a portion of therapy sessions can occur virtually with caregiver assistance. Although research examining telehealth in rehabilitative therapy is limited, hybrid therapy regimens combining in-person and virtual sessions can be superior to in-person sessions alone for treatment of musculoskeletal conditions [33, 34]. Incorporating virtual sessions can also help reduce the burden of traveling and allow greater schedule flexibility. Given the rarity of AADC deficiency, telehealth visits allow access to experts with experience treating patients with the disorder, regardless of location. This access is beneficial considering the severity of patients with AADC deficiency and difficulties of transporting such medically fragile children. In addition to increasing access to experts, virtual therapy sessions with the patient and caregivers are helpful to ensure compliance with the home exercises. Finally, the coaching models used in telehealth visits create a collaborative relationship between therapists and caregivers and may also increase caregivers’ sense of empowerment in caring for their child [33].

#### Therapy to facilitate active movements

After gene therapy, as the enzymatic pathway has been restored, patients will likely demonstrate increasing spontaneous active movements, both in amplitude and frequency, progressively during the early phase. However, improvement induced by gene therapy alone may vary among patients. As with motor development in infancy, spontaneous active movements may serve as the foundation for patients identifying appropriate muscle actions for voluntary, coordinated movements and adapting for future functional use [35]. As a result, facilitating typical patterns of active movements should be a major focus of therapy during the early stage following gene therapy.

Facilitating typical patterns of active movements in therapy can be accomplished via the combined use of manual work from the therapist and movements from the patients, as well as via proper positioning of the body to allow the patient to perform spontaneous active movements more easily. Developmentally supportive positioning in supine promoting muscle activation and antigravity control of proximal flexors is strongly recommended for patients with truncal hypotonia persisting in the early stages after gene therapy. Aquatic therapy, where therapeutic exercises are performed in a pool, and providing additional support such as using a gait trainer, are also highly recommended to facilitate active movements. Further explanation is provided in the *Supplemental Forms of Therapy* and *Assistive Technology* sections.

In addition to maintaining static posture, post-treatment physical therapy should focus on dynamic activities of posture transition, critical for active exploration and development of more advanced locomotion skills. It is worth noting that as patients receive gene therapy at an age closer to 2 years and beyond, factors including neuroplasticity and body dimensions will pose additional challenges compared to typically developing infants in gaining the ability to transition postures. With movement repertoires gradually appearing after gene therapy, therapists should carefully observe the emergence of new movements and guide patients to apply or adapt these movements for appropriate posture transitions.

**Fig. 2:**
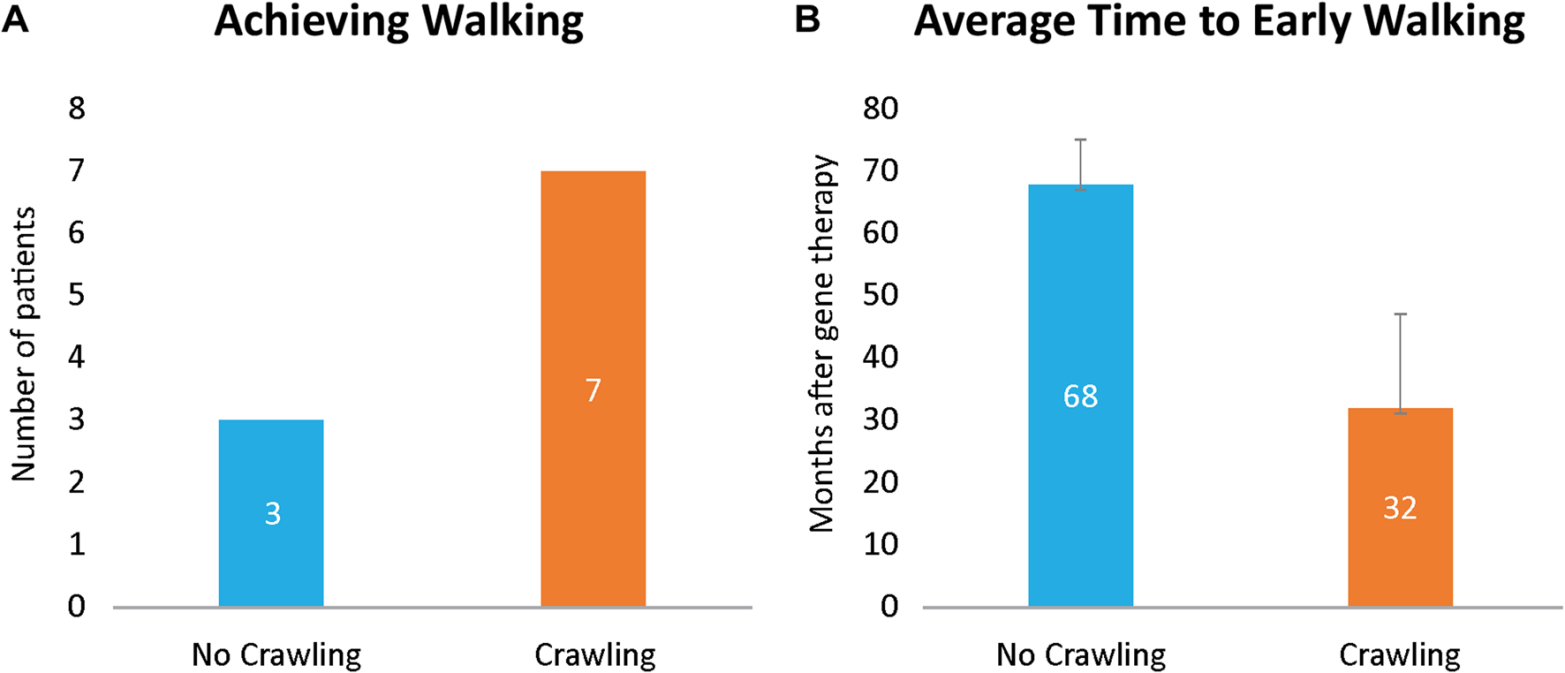
**a** Number of patients who achieved walking based on crawling status. **b** Average time to early walking based on crawling status. Data from crawling and walking items of the locomotion subtest in PDMS-2 of patients who were followed up over 2 years after gene therapy was included. Error bars represent standard deviation

For instance, once the patient can sit with propped arm support, early ambulation training should be incorporated. Once the patient can take some steps, regardless of crawling ability, further training is needed to achieve proper reciprocal stepping necessary for development of functional ambulation. However, this is not to downplay the importance of learning to crawl. Although typically developing children may skip crawling and progress straight to walking, crawling is an important locomotion milestone and should not be overlooked in the post–gene therapy physical therapy program [36]. In our clinical experience, patients who develop the ability to crawl (moving around on hands and knees with the belly off the floor) demonstrate independent walking sooner than those who do not crawl. Patients who do not crawl have more difficulty developing appropriate core stability and mastering the ability to coordinate their body segments for proper balance, a critical factor for independent walking. Data from 21 patients in two of the clinical trials showed that among patients who developed walking ability, the number of patients who crawled was more than twice the number of patients who did not crawl, and the average time of the emergence of independent walking was 32 and 68 months after gene therapy for patients with and without crawling ability, respectively (Fig. 2a, b). More specifically, based on these data, patients who crawled were able to develop walking 3 years earlier in general than those who did not crawl.

#### Age-appropriate experiences and functional use of active movements

**Fig. 3:**
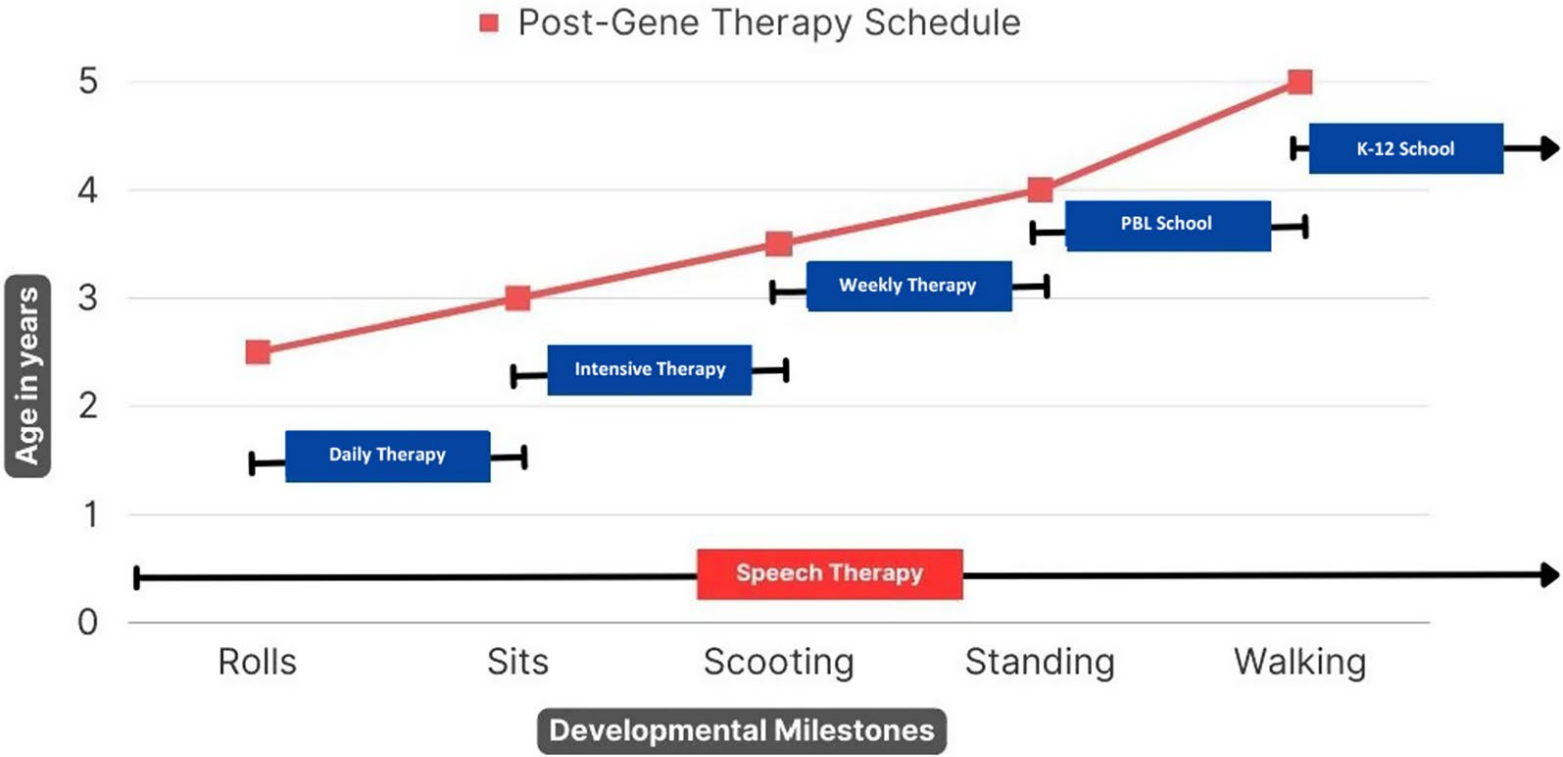
An example of real-world experience of post-gene therapy rehabilitation schedule in one patient receiving eladocagene exuparvovec. The patient immediately began OPT in conjunction with traditional speech therapy continuously. Daily PT for head control consisted of 1 h of core exercises for 2 weeks. Intensive therapy for sitting independently was for 2 h daily for 1 month. Weekly therapy for head control, trunk strength, and scooting independently included daily speech therapy and 4 h of PT and OT for 3 months. Play-based learning focused on standing independently at PBL school: 5 days a week, 4 h daily, for 1 year, with supplemental PT 3 times a week for 1 h. Walking was the focus at the K-12 school: 5 days a week, 8 h daily, with learning support 3 days a week, OT twice a week, PT once a week, and speech therapy once a week, with the option to modify as needed

With severe impairments across all developmental domains since infancy, patients with AADC deficiency have limited age-appropriate experiences strongly impacting the advancement in development. In addition to training focused on providing ability-appropriate activities, therapy properly incorporating age-appropriate experiences in aspects of motor, sensory, language, cognition, self-care, and social interaction is recommended (Fig. 3.). It is important that patients, particularly after receiving gene therapy, are provided age-appropriate experiences to promote and encourage context-related functional use of active movements, as these can aid in improving abilities of various developmental domains. For instance, patients should be exposed to various postural experiences, such as being placed in prone or supported sitting positions, before independently transitioning to these positions. Similarly, patients should experience standing using a stander or other support before they transition to standing independently. Ideally, this type of standing experience should occur daily for > 60 min. In addition to postural experiences, object manipulation with a variety of toys provides another opportunity for age-appropriate experiences incorporating active movements. As patients develop “hand-to-mouth” ability following gene therapy, maximizing the opportunity for object manipulation and other hands-on experiences can effectively help prevent this from becoming a problematic behavior.

**Fig. 4:**
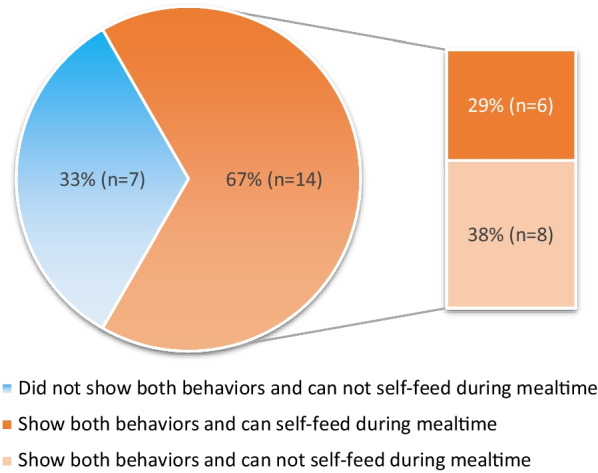
Proportion of patients able to carry object to mouth, show persistent reaching behaviors and/or able to self-feed. Data of two items (carries object to mouth and persistent reach) from Bayley-III and current self-feeding ability of twenty-one patients who were followed up over 2 years after gene therapy were included

As patients gradually gain movement repertoires following gene therapy, practicing and maximizing available active movements in a functional context is important for further motor skill development. This allows the patient to practice their motor skills by incorporating them throughout their day rather than solely practicing these movements in therapy sessions. Based on our experiences, an inability to apply active movements for functional uses, or activities of daily living (ADLs), is one of the major limitations that prevent further advancement in development. ADLs usually do not occur easily or spontaneously in patients with AADC deficiency following gene therapy. For instance, data from 21 patients in two of the clinical trials showed that over half of the patients who displayed both the behaviors of carrying an object to their mouth as well as persistent reaching for objects still did not achieve self-feeding. Despite these skills being achieved on average 12 months after receiving gene therapy (range of time to develop skills: 6–24 months), more than half of these patients did not develop self-feeding ability years after these two behaviors presented (Fig. 4). This disconnect between active movement achievement and functional use for ADLs highlights that extra efforts and guidance are required to facilitate these transitions. Early and continuous practice of applying active movements for functional use is recommended and is critical for developing independence. For instance, participating in self-feeding once hand-to-mouth ability develops, engaging transfer in standing once the ability of weight-bearing of lower extremities emerges, or starting toilet training once the ability of independent sitting appears.

#### Incorporating play into rehabilitation

Incorporating play into rehabilitation takes advantage of a child’s inclination to play and natural learning that occurs during play to aid motor development [17, 18]. Playing provides patients with age-appropriate experiences and exposure to a variety of objects with which they can physically interact [37]. As the patient is focused on playing, an enjoyable activity, they become less focused on the motor demands of the activity and more motivated and engaged with the activity. This allows the therapist or caregiver to guide them toward appropriate movements while playing [37, 38]. Another benefit of using play to engage motor skills is that play is free and can be easily incorporated into the patient’s day in a number of ways using a range of toys and environments. The addition of play to conventional rehabilitation has been shown to improve hand function compared to conventional rehabilitation alone in children with CP [38]. The benefits of play extend beyond motor development; play can reduce stress, improve self-confidence, and promote creativity, emotional regulation, problem-solving, and language development in children [17, 18]. As patients with AADC deficiency may lack motivation and play experiences compared to typically developing children, the advantages of incorporating play into rehabilitation motivate patients to practice active movements and provide an opportunity for direct functional uses, as additional guidance and practice are often needed for patients to apply and adapt movements for functional activities following gene therapy.

#### Sensory integration therapy and sensory-based intervention

Sensory integration therapy (SIT) and sensory-based intervention (SBI) use play-based sensorimotor activities to improve sensation processing and integration. Though the methods utilized may vary, SIT/SBI most frequently focus on stimulating vestibular, proprioceptive, and tactile sensory systems [39, 40]. Activities used in SIT/SBI include walking on tactile walkways, tracing the body, balancing on a balance board, identifying the weight of unknown objects, applying pressure to different body parts, and more. The incorporation of vestibular stimulation into post-treatment rehabilitation programs, including rocking, spinning, rolling, or swinging, which most patients with AADC deficiency enjoy and benefit from, is highly recommended. Patients with CP receiving forms of SIT/SBI reported improvements in gross motor skills as well as sensory integration and balance, communication abilities, and attention [40]. Benefits of SIT have also been shown in patients with succinic semialdehyde dehydrogenase deficiency, a neurotransmitter disorder with symptoms including hypotonia and poor motor control. Following addition of SIT to regular rehabilitation regimens, patients gained motor abilities, increased sensory awareness and tolerance for movements, and showed improved cognition [39].

#### Assistive technology

A range of assistive technology should be incorporated into the post-gene therapy physical rehabilitation regimen. Devices such as ankle-foot orthotics, positioning chairs, standers, gait trainers, or wheelchairs are important for patient progress and can help patients achieve proper positioning to facilitate movement. Many patients with AADC deficiency are non-ambulatory and at risk of complications from extended time in seated or lying postures before gene therapy. Standers can be particularly helpful by improving weight bearing and range of motion, providing pressure relief, increasing psychological well-being, and more [41], and may be especially beneficial in patients already capable of standing with some support. It is recommended that a stander with ankle-foot orthotics be used gradually for at least 60 min per day, 5 days per week. However, standers should only be utilized in patients able to be positioned properly within the stander, as patients not able to achieve proper positioning may not receive appropriate support beyond the mid-torso level.

Assistive devices should be prescribed based on functional and caregiver goals, with this adaptive mindset employed regardless of whether a patient has received gene therapy treatment, but for those who have, it should be exercised throughout the post-gene therapy journey. Since motor abilities are expected to improve following gene therapy, assistive devices that enable body segments to move more freely are important to aid in development of voluntary active movements. For example, gait trainers or walkers with trunk and pelvic support can help facilitate stepping and weight-bearing ability. Once the patient can sit with propped arm support with or without the ability to bear weight through their legs, ambulation training with a gait trainer or walker with trunk and pelvic support can be added to the rehabilitation program to promote further development of motor milestones. More advanced assistive technology such as powered lower limb exoskeletons may also be useful for ambulation training, as these have shown positive effects in patients with CP [42].

Most patients will also require a custom medical stroller or wheelchair during this time to accommodate head, trunk, and limb positioning for long distances. Depending upon motor and cognitive improvements, patients may be able to drive a power wheelchair. As the patient grows, they will likely become too heavy for caregivers to transfer without support. At this stage, a mobile lift can help transfer the patient at home, if the child is yet unable to bear weight to aid in mutual transfers. Cultural differences in the use of assistive devices should be taken into consideration by the physical therapist and treatment team. For instance, in cultures where wheelchairs are not frequently utilized by pediatric patients, an adaptive stroller that still enables proper positioning may be a better option if it means the patient and their family will utilize it more frequently. As children treated with gene therapy gain new motor skills and grow, frequent assessment of equipment fit and need will be required.

### Supplemental forms of therapy

Supplemental forms of therapy, such as aquatic, music, and hippotherapy, have positive effects in patients with movement disorders [15, 16, 19, 20, 38–40, 43, 44] and may help improve outcomes after gene therapy in patients with AADC deficiency. As an added benefit, these types of rehabilitation can be very enjoyable, which may improve patient engagement with and motivation for practicing motor activities. Given the rarity of AADC deficiency, evidence supporting use of supplemental therapies in patients with the disorder is limited. Therefore, patients and caregivers should work with their therapists and physicians to identify the most appropriate methods to incorporate into the treatment plan.

#### Aquatic therapy

Aquatic therapy, a method where therapeutic exercises are performed in water, takes advantage of buoyancy to give patients the most autonomy possible in movements and overcome land-based motor limitations. Water is a low-impact environment for mobility; patients can experience freedom to move around in a pool without fear of falling. Many patients who received gene therapy can perform actions and movements in this unique environment that cannot be easily accomplished on land; the sense of freedom to move motivates patients to repeat actions and movements naturally and spontaneously. Specifically, in a pool, less effort is required for patients to carry out reciprocal leg movements essential for locomotion. Additionally, water viscosity provides multi-dimensional resistance to help improve muscle strength while hydrostatic pressure helps strengthen respiratory muscles and increase blood and oxygen flow to muscles and the brain [20, 45]. In children with Rett syndrome, aquatic therapy has been especially beneficial for active movement training in patients not strong enough for movement therapy on land, allowing them to move through their available range of motion more freely in the water. In addition to physical improvements, these patients showed improvements in mood and relations with family and schoolmates, revealing the broader impact of aquatic therapy for patients with neurodevelopmental disorders [20]. Aquatic therapy also enables caregivers to provide better physical support in water as they are carrying less weight, ultimately increasing comfort with in-home land therapy.

#### Hippotherapy

Hippotherapy utilizes rhythmic movements of a horse to provide repetitive challenges to a patient’s balance, posture, and strength while helping integrate sensory input with motor responses. The therapist can target different sensorimotor skills and postural control by having the patient sit or move on the horse in various positions. The moving horse also provides rapidly changing conditions in a short period of time that the patient must adapt to using trunk stability and other motor reactions. Hippotherapy has been effective in children with CP, where improvements in trunk control and head stability were observed following 12 weeks of hippotherapy. Hippotherapy is also linked to improved self-confidence, social interactions, and overall quality of life in these patients [15]. Improvements in gross motor function, including head and trunk control, have also been observed in children with Down syndrome following a hippotherapy regimen [44]. As patients with AADC deficiency show hypotonia especially around the core even after gene therapy, hippotherapy provides an entertaining alternative to strengthen core muscles. Examples of exercises involved in hippotherapy include transitioning between positions, riding without holding on with their hands, and handing objects back and forth or catching or throwing a ball with the therapist [15, 44].

#### Music therapy

Music therapy takes advantage of music’s ability to connect motor and cognition while facilitating learning and emotional experiences [43]. The structured timing of rhythm-based activities can also help patients develop the ability to coordinate and synchronize movements [16, 43]. One type of music therapy that integrates active movements with music and rhythm is therapeutic instrumental music performance (TIMP). In TIMP, patients aim to play musical instruments using physical actions that mirror other functional movements. Instruments are deliberately placed in different positions relative to the patient to improve range of motion, limb coordination, and functional movements [16]. In children with severe bilateral CP, this music therapy resulted in improved upper-limb function, suggesting that patients with AADC deficiency may also experience similar benefits [43].

In addition to motor improvements, music therapies such as TIMP can also increase patient participation and motivation, two critical factors in therapy success [16, 19]. Integration of movement therapy with music and rhythm can also make active movement therapy more enjoyable for the patient [19]. Singing has been incorporated into aquatic therapy programs of children with Rett syndrome to increase patients’ enthusiasm [20]. As many patients with AADC deficiency demonstrate special interest and respond well to songs, rhymes, beats, and rhythms after gene therapy, the evidence provided by patients from other diseases further illustrates the potential of music-based therapies to supplement physical therapy in children with AADC deficiency.

### Cognitive and communication training

There is a clear interplay between early cognitive and motor development, and given the link between cognitive processing and motivation [46, 47], it is possible that development of motor milestones may be further impaired by delayed cognitive development. Even after gene therapy, many patients do not have previous motor experience and social involvement to draw from to form a new skill. This, combined with clinical trial evidence demonstrating improvements in cognition and language are possible following gene therapy [13], highlights the importance of focusing on cognitive function and development, which play a critical role across the post-treatment physical rehabilitation journey. Certain strategies can be employed before or after gene therapy to nurture cognitive development and help patients develop awareness of their body, align body and mind, evoke their interests to interact with objects and environments, and ultimately facilitate motivation and maximize outcomes throughout their journey. Importantly, parent-child interactions rooted in daily activities also have a strong influence on cognitive and communication development.

Training focusing on optimizing visual fixation and object tracking are often overlooked but are extremely critical, as based on our experiences, most patients are highly interested in faces but pay no attention to toys, even after gene therapy, if not properly trained. Providing patients with age-appropriate, rather than strictly ability-appropriate, experiences can also help improve cognitive development. This can involve toys, books, play activities, and incorporating rhythm into body movement exercises through children’s songs or musical instruments. For patients with limited voluntary movements, these age-appropriate experiences can be provided through guidance or modeling. The goal is to provide the child with increasingly challenging activities to further promote cognitive development, but attention should be paid to incorporating age-appropriate stimulation to various degrees.

A focus on speech and communication training is also critical to post-treatment rehabilitation success, as it may enhance development of cognition and patient comprehension of instructions during therapy. For instance, Augmentative and Alternative Communication (AAC) methods may enable effective communication between patients and their treatment team and caregivers [48]. It is recommended that AAC be introduced early in the post-treatment rehabilitation journey. In addition to oral language skills, speech and communication therapy should focus on non-oral communications such as facial expressions, eye contact, gestures, and body movements. Similarly, Oral Placement Therapy (OPT), a speech therapy methodology combining auditory, visual, and tactile stimulation, may also be a beneficial supplement to a speech therapy program. As speech is a tactile-proprioceptive act, tactile and proprioceptive elements of OPT may be particularly beneficial for patients with motor impairments, who cannot use more traditional auditory or visual input to produce or imitate speech sounds [49].

Moreover, speech therapy plays a critical role in improving other oral functions such as chewing, swallowing, and other feeding skills, usually impaired to various degrees in patients with AADC deficiency [4, 5, 50].

### Additional considerations for developing a post-gene therapy treatment program

#### Public therapy services

Therapy costs can be a tremendous financial burden on caregivers, especially as physical therapy is only one component of caring for a child with AADC deficiency [51]. Therefore, families should leverage local government funding, benefits, and resources, including publicly funded therapy services, to help reduce financial burden. Forms of publicly funded therapy services are available in several regions for children with developmental delays or other disabilities. For patients diagnosed and treated with gene therapy prior to age 3 years in the United States, early intervention services can be utilized. Patients aged ≥ 3 years may be eligible for therapy services through their local public elementary school [52]. Early intervention services for children from birth to school entry and therapy services for older children with developmental delays and other special needs are also available throughout Canada [53–55]. In Taiwan, children diagnosed with developmental delay are eligible to receive therapy services, including occupational, physical, and speech therapy, supported by early intervention government funding [56]. While therapists in these services have extensive experience in pediatric therapy and movement disorders such as CP, they will likely have limited or no experience treating patients with AADC deficiency. Therefore, the patient’s physician should regularly communicate with the therapist and provide guidance.

One limitation is the frequency of the sessions, which may not be sufficient for patients with AADC deficiency. Given the extensive rehabilitation needs of patients following gene therapy, private therapy should be used to supplement these sessions and provide more intensive training as needed.

#### Dyskinesia

Following treatment with gene therapy, patients may experience dyskinetic events. These involuntary movements are transient and likely the result of dopamine receptor hypersensitivity due to long-term dopamine deficiency resulting from AADC deficiency [13]. Presence of post-treatment dyskinesia should not delay the start of rehabilitation. In fact, these involuntary movements of body segments during dyskinesia episodes can provide an important foundation for future development of functional movements, similar to spontaneous movements during early infancy [35, 57].

If dyskinesias occur, a number of techniques may be utilized to manage movements and prevent therapy interference. Use of weight, via compression vests, weighted blankets, or ankle weights has been beneficial. Therapeutic techniques and guidance for producing optimal movement patterns can also be utilized during this period. Finally, proprioceptive input to help activate joints and muscles can be added to the rehabilitation routine for increased body awareness. Dyskinesias may also impact coordination of breathing and swallowing, although this was not observed in the eladocagene exuparvovec trials. Therefore, swallowing assessments and consultations on feeding skill adjustments are recommended to promote safety while eating. This may involve oral sensitization/desensitization massage, positioning adjustment, tableware adjustment, and food texture adjustment.

#### Regular orthopedic follow-up

Regular orthopedic follow-up with hip and spine assessments are important to check for development of scoliosis and contractures, especially as patients approach pre-puberty. Monitoring for hip dysplasia or subluxation is important in non-ambulatory children, and an annual pelvic radiograph is recommended. Additionally, hypotonia or dystonia in AADC deficiency can contribute to development of hip subluxation. As such, hip radiographs should be obtained if the patient develops pain with or without hip movements or asymmetrical hip abduction [58]. However, these types of complications from the underlying disorder do not typically impact the ability to implement and conduct therapy.

#### Cross-functional collaboration following gene therapy

Management of AADC deficiency requires a multidisciplinary team, including physical therapists, speech and language therapists, occupational therapists, social workers, dieticians, and physician specialists [4]. Collaboration between members of the treatment team when devising a post-treatment plan is critical; physical therapy should occur in parallel with speech therapy, occupational therapy, and regular follow-up visits with the pediatric neurologist or other specialist primarily leading care. Continued coordination and collaboration throughout the post-treatment journey will help ensure each patient receives rehabilitation regimens most appropriate for their abilities at every stage and that patients and families have access to the necessary programs, facilities, and equipment.

The role of the treatment center and referring physicians in patient care evolves across the post-gene therapy journey. Prior to discharge from the treatment center, physicians should help advocate for post-treatment therapy for their patients and connect patients and caregivers with available resources. If patients can receive therapy in an inpatient rehabilitation center, physicians should meet with the patient, family, and therapists prior to discharge to prepare and align on next steps for rehabilitation. Communication between the inpatient team, whether at the treatment center or an inpatient rehabilitation center, and the patient’s local physical therapists and other providers is critical at this stage to adequately prepare the local treatment team for the patient’s return home. Once discharged, physicians should also monitor for treatment side effects, including dyskinesias that may interfere with physical therapy if not properly managed.

### Caregiver involvement in post-treatment therapy

As discussed, the importance of parental and caregiver support, involvement, and commitment to rehabilitation is crucial for post–gene therapy improvements. Parents and caregivers must recognize that the potential for developmental improvements following gene therapy may be restricted if the patient does not follow the proper post-treatment therapy plan. Moreover, as patients demonstrate basic motor abilities and exploratory behaviors, caregivers should provide opportunities for their child to continue exploring and applying these abilities as often as possible within safe limits. The treatment team, including therapists, can help support caregivers and encourage their involvement and enthusiasm in their child’s treatment plan by openly communicating with them throughout the post-treatment journey. This enables caregivers to better understand their child’s progress and know what is expected from them to best help their child. Therefore, parent/caregiver empowerment should be a top priority for the treatment team when working with patients with AADC deficiency.

In addition to regularly and openly communicating with caregivers, therapists should continuously coach them on how to properly position and interact with their child as their motor abilities change and encourage them to practice at home through functional activities. Because costs may restrict frequency with which therapy sessions occur, caregiver-mediated practice can allow the patient to practice new motor skills outside of their regular therapy sessions and can further increase mobility and motor development. In addition to the suggestions in the earlier in-home therapy section, some caregivers may find specific therapist-assigned “homework” for working with their child outside of therapy sessions motivating and beneficial [31].

While patients likely relied on their caregiver for all daily living tasks, this may begin to change as their motor function improves following gene therapy. Caregivers should be encouraged to allow their child the freedom to make functional gains and experience more autonomy as they progress on their post-treatment journey. As an example, they can allow their child to attempt self-feeding once hand-to-mouth ability develops, rather than being fed exclusively by the caregiver. While caregivers may initially struggle to allow their child the independence required to make such gains, therapists and the treatment team should continue to emphasize the importance of reinforcing empowerment, not helplessness, by promoting child-initiated movements in their communications with caregivers.

Caregivers also play a large role in helping their child develop cognitive and communication abilities. They can support this development by describing the actions or activities that they or their child performs by observing, interpreting, and reacting to their child’s responses in a supportive way and by providing opportunities for their child to make decisions as often as possible to strengthen their child’s desire to communicate and promote autonomy.

Caring for a child with AADC deficiency has been shown to place a financial, mental, emotional, and physical burden on caregivers [51], with one survey of patients with AADC deficiency and their caregivers finding that 71% were fully dependent on their caregiver [7]. To help reduce the financial burden, social workers, physicians, and therapists can help connect caregivers and families to publicly funded therapy programs and other resources to help obtain necessary assistive devices or home modifications to help their child progress [59, 60]. Social workers can also help connect caregivers to support groups and other networks to help reduce stress and ease the mental and emotional burden [4, 60]. Similarly, supplemental therapies like cognitive play, aquatic therapy, and hippotherapy may not only be enjoyable for the patient but can also provide a bonding experience for the family, allowing them to make memories together while participating in activities their child enjoys.

While factors correlating with response to gene therapy have been identified [13], many unknown variables likely contribute to treatment response in patients with AADC deficiency. Though everyone involved in the patient’s care hopes for the best possible results following gene therapy, the treatment team should also prepare caregivers for the range of outcomes that may occur and what improvements are realistic for their child.

### Recommendations for post-treatment physical therapy regimen

Though the post-gene therapy journey will differ among patients, information and experiences highlighted throughout this article lead us to propose the following summary of therapy recommendations for patients with AADC deficiency, with the caveat that specific rehabilitation goals and programs will depend on individual patient ability and needs. While muscle overexertion may be a concern for patients with degenerative neuromuscular disease [61], AADC deficiency is not a progressive disorder [62]. As such, once neurotransmitter biosynthesis is restored following gene therapy, although patients may easily fatigue both physically and mentally, they should be continuously challenged to make gains during therapy without fear of muscle overexertion, while monitoring fatigue and developing a reasonable practice schedule. Given that patients with AADC deficiency have poor endurance in general and are still prone to hypotension and hypoglycemia after gene therapy [13], training sessions with tasks carried out in shorter duration, higher frequency, and with proper rest is recommended.

**Fig. 5:**
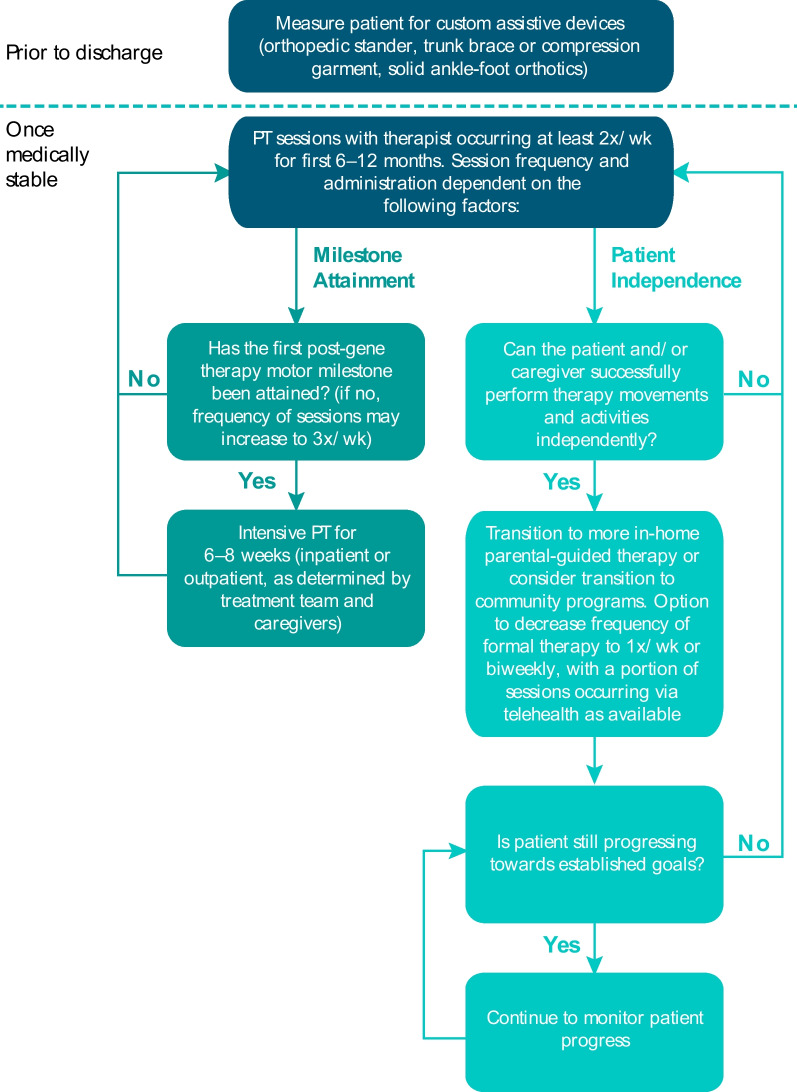
Evaluation flow chart for determining post-treatment physical therapy session frequency in the first 12 months following gene therapy

The recommended physical therapy frequency for patients with AADC deficiency following gene therapy was developed in line with the guidelines established by Bailes et al. This publication recommends 4 factors to guide therapy frequency: (1) patient’s ability to participate and benefit from the therapy process, (2) presence of a critical period for rapid recovery, (3) amount of therapist expertise required, and (4) level of family support needed to meet therapy goals [63]. Briefly, physical therapy sessions should occur more frequently during initial stages of learning a skill or movement, or during critical periods when milestones are more likely to develop. As the patient can perform the movement with little therapist guidance, frequency and/or intensity of physical therapy sessions can decrease (Fig. 5) [63, 64].

Rehabilitation should begin as soon as patients are medically stable following neurosurgery. Weekly follow-ups should take place for the first 4 to 6 weeks following treatment to help physicians monitor for treatment side effects, including dyskinesias that may cause safety issues if not appropriately managed. Physical therapy should occur at least twice per week for the first 6 to 12 months following gene therapy. Once therapy activities and movements can be performed by the patient and/or caregiver, therapy session frequency can be reduced to weekly or biweekly, and a portion of sessions can occur via telehealth as available, as long as progress toward established goals are met. Therapists should collaborate with caregivers to develop an in-home therapy program so patients can practice and advance their motor development outside of therapy.

Attainable goals following gene therapy include milestones such as head control, sitting with and without assistance, standing with and without support, and walking with and without assistive devices. Intensive physical therapy, whether inpatient or outpatient, should occur once the first motor milestone is attained, but may also be considered if the first motor milestone has yet to be attained. This may involve a period of 2 weeks in an inpatient pediatric rehabilitation unit if possible, or an increase in session frequency for patients unable to access inpatient therapy. This increased frequency should continue for 6 to 8 weeks to take advantage of what appears to be a critical period for further milestone attainment following attainment of the first milestone based on available data [27].

Direct therapy for achievement of motor milestones for many patients can be conducted through floor-based exercises with proper use of assistive devices. Therefore, before discharge from the hospital following gene therapy, patients should be measured for a custom orthopedic stander (with head support if required), trunk brace or compression garment, and bilateral solid ankle–foot orthotics. A pediatric physical therapist and equipment vendor should then conduct an in-home assessment to ensure the equipment fits the patient appropriately and caregivers can properly use equipment at home.

Before development of sitting with propped arm support, rehabilitation activities should include positioning, facilitating active movements, promoting head and trunk control, standing with the assistance of a stander, and supplemental therapies such as aquatic therapy. As movements for functional activities emerge, physical therapy should focus on promoting and maximizing context-related functional use of these voluntary movements. For example, once the patient can bring their hand to their mouth, self-feeding should be encouraged and practiced. Additionally, once the patient can sit with propped arm support, early ambulation training should be introduced. Finally, cognitive and communication training as well as exposure to age-appropriate experiences should occur throughout the post–gene therapy journey.

As motor improvements have been observed beyond 5 years following treatment with eladocagene exuparvovec [13], continued rehabilitation is essential for maximizing patient improvements. Physical therapists should consider an episodes-of-care approach for long-term physical therapy, with alternating periods of therapy and breaks in therapy. Length and intensity of an episode of care should be determined by the treatment team depending on patient and family goals and will likely vary depending on skill or movement [64, 65]. Established assessment scales for motor, cognition, speech, and other symptoms should be utilized in order to accurately track post-gene therapy progress 66. In addition to tracking patient progress over time, the use of standardized assessment scales can minimize effects of bias resulting from clinical expectations, can assist the treatment team in planning interventions, and may help determine patient eligibility for early intervention services [67, 68]. Given the rarity of AADC deficiency, patients and caregivers should also be encouraged to enroll in patient registries. These registries will provide valuable insights into the real-world impact of gene therapy and post-treatment therapy regimens on patient development, allowing for an improved understanding of post-treatment therapy approaches to maximize patient improvement.

## Conclusions

The approval of eladocagene exuparvovec, the first gene therapy indicated for treatment of AADC deficiency [10], expands the range of motor and cognitive outcomes for patients with the disorder. Following treatment, achievement of motor milestones such as sitting, standing, and even walking are possible [13, 27]. However, a more holistic perspective is key to designing a therapy regimen following gene therapy treatment, specifically one tailored to the varying needs of patients to help maximize post-gene therapy improvements. As eladocagene exuparvovec was only recently approved, there is currently a lack of therapy guidelines for this post-treatment period. This is an especially critical unmet need given the rarity of AADC deficiency, as most therapists will have little to no prior experience treating these patients.

In this article, we have discussed several considerations for post-treatment therapy approaches and proposed recommendations that we believe caregivers, therapists, and the wider treatment team will find valuable as they develop individualized post-treatment therapy plans for their patient. Given the breadth of impairments experienced by patients with AADC deficiency, a post-treatment therapy plan should incorporate a variety of approaches to achieve the best possible outcomes. This includes a focus on cognitive and communication training, active movement training, periods of intensive rehabilitation timed with milestone achievement, incorporation of therapy exercises and activities at home, and supplementing with forms of therapy that the patient may find more enjoyable and engaging, such as aquatic therapy or hippotherapy. In addition to these specific recommendations for the focus of the therapy program, post-treatment therapy plans need to include an emphasis on caregivers, as they play a crucial role in all aspects of their child’s post-treatment life. Programs should be developed in collaboration with caregivers as much as possible to improve feasibility, confidence, and compliance. Caregivers should also be connected with financial, emotional, and mental support throughout the post–gene therapy journey to help promote their well-being and quality of life.

Outside of AADC deficiency, the role of therapy following treatment will be increasingly important to consider for other rare diseases as gene therapies and other treatments are developed and approved for these disorders. We believe that the considerations and recommendations presented here will also prove beneficial beyond the AADC deficiency community.

## Data Availability

Not applicable.

## Abbreviations

AAC: Augmentative and alternative communication
AADC: Aromatic l-amino acid decarboxylase
AIMS: Alberta infant motor scale
Bayley-III: Bayley scales of infant and toddler development–3rd edition
CP: Cerebral palsy
OPT: Oral placement therapy
OT: Occupational therapy
PBL: Play-based learning
PDMS-2: Peabody developmental motor scales–2nd edition
PT: Physical therapy
SBI: Sensory-based intervention
SIT: Sensory integration therapy
TIMP: Therapeutic instrumental music performance
